# Comparison of pre‐hospital triage training by role playing and lecture on nursing students' knowledge, attitude and performance

**DOI:** 10.1002/nop2.464

**Published:** 2020-04-21

**Authors:** Hamid Heidarzadeh, Zeinab Heidarzadeh, Arman Azadi

**Affiliations:** ^1^ Department of Nursing Faculty of Nursing and Midwifery Ilam University of Medical Sciences Ilam Iran; ^2^ Emam Ali Hospital Ilam University of Medical sciences Ilam Iran

**Keywords:** lecture, nursing students, pre‐hospital triage, role playing, training methods

## Abstract

**Aim:**

The objective of this study was to determine and compare the effectiveness of two methods of role playing and lecture on knowledge, attitude and performance of nursing' students in the context of pre‐hospital triage.

**Design:**

This was a pre‐test–posttest quasi‐experimental study.

**Methods:**

A total of 66 nursing students (third year) were assigned to two groups, the control group (*N* = 23) and intervention group (*N* = 23). START pre‐hospital triage was taught to two groups by using a lecture (control group) and role playing (intervention group) method. Immediately before the intervention and 4 weeks after the training, students' knowledge, attitude and practice in both groups were assessed through a questionnaire and a checklist. Data were analysed using SPSS software version 21.

**Results:**

The results showed that the mean scores of knowledge, attitude and performance increased after intervention in both groups (*p* < .05). The mean (*SD*) difference of total performance score from baseline to follow‐up in the experimental group and the control group was 23.91 (13.83) and 7.00 (13.20), respectively (*p* < .001). While there was no significant difference between the mean (*SD*) difference of knowledge and attitude scores in the experimental group and the control group before and after the intervention (*p* > .05).

## 1 INTRODUCTION

The word triage is derived from the French word trier, which means to sort or select. Triage is used for two or more injured persons and first aid should be used for a single injury. Triage is divided into two pre‐hospital and in‐hospital triage (Drobatz, Hopper, & Rozanski, 2018). The main reasons requiring triage can largely determine the philosophy of triage. The three main reasons the need for triage are as follows: incompatibility between existing facilities and needs, especially in the field of human resources (physician, nurse, etc.) and equipment, the presence of many injured people and patients in a certain time (such as accidents, unpredictable events or overcrowding of patients in emergency departments) and the attitude and perception of people in emergency and non‐emergency cases. In light of the preceding, it seems that the purpose of triage is to make optimal use of the resources and existing facilities (Ebrahimi et al., 2016).

To rescue the injured people of a crisis, the use of all facilities and existing capacities within the first 72 hr after a crisis is of high importance. So, the role of nurses by quick attendance and urgent care provision and injured people transfer is crucial. Hence, designing theoretical and practical courses to train specialist nurse—crisis should be at the priority planning (Atashzadeh shorideh, Nikravan Moofrad, & Zohri Anbouhi, 2007). Nurses are the largest groups among medical staff that offer services in a critical setting and given that today's students are tomorrow nurses, their preparedness can be crucial in the face of unpredictable events (Bahrami, Aliakbari, & Aein, 2014; Khajeahmadi & Jahanpour, 2017). Most skilled nurses in the daily cares of patients in hospital inpatient ward can make an appropriate decision, but due to unpredictable events, a crisis setting may affect the performance and clinical judgment of nurses. Moreover, because of stressful position and considering the priority of caring the injured people in an acute and critical setting, there is no enough time to gain experience, so nurses' preparedness for optimal performance at times of crisis is crucial (Heidarzadeh et al., 2017; Lyles, 2009).

## 2 BACKGROUND

Many studies show that knowledge or performance concerning triage is not desirable in Iran and throughout the rest of the world (Aghababaeian et al., 2019; Heidarzadeh et al., 2017; Sedaghat et al., 2012; Tuyisenge et al., 2014). Nurses' poor knowledge regarding triage can arise from the absence of sufficient training during both study and work periods (Mahmoodian, Eghtesadi, Ghareghani, & Nabeiei, 2016). Training is the foundation of learning types and today in educational planning, education and improved human resources can be thought of as a strategy for increasing human capital and positive consistency with circumstance change (Frenk et al., 2010).

In this regard, training methods are divided into traditional and modern methods; traditional methods (such as lecturer) are teacher‐centred, and modern methods (such as role playing) are the student‐centred (Maddry et al., 2014). Bloomberg classifies behaviours into cognitive, emotional and psycho‐emotional domains, which can be experienced simultaneously. There are a variety of training methods and tools for cognitive abilities development. Lecture method is applied as a unique and complete method in training cognitive behaviours, but it is not desirable for levels above the cognitive domain (Liebrecht & Montenery, 2016). The lecture method is not able to involve students in learning and problem‐solving principles, but simulation‐based learning is more effective in students lifelong learning because of creating a more actual position and the possibility of repeating positions. Simulation ideally can provide a clinical position with diverse complexities that enable the student to make a decision, prioritize problems and choose the best solutions. Simulation's advantages include making a mistake without harming a patient, active learning, an opportunity for immediate feedback, the control of learning environment, enhanced critical thinking and problem‐solving, timing of designed learning and interference and manipulation in a learning environment (Bux, 2009; Sabounchi, Sabounchi, & Safari, 2019). However, cases such as cost, skill and educators' experience about modern methods have confronted the use of this with challenges (Naderifar, Ghaljaei, Jalalodini, Rezaie, & Salar, 2016).

Simulation as one of the ways of modern training has different types. Acharya, Shukla, Acharya, Vagha, and Vagha (2014) considered role playing as one particular type of simulation that focuses on the interaction of people with one another (Acharya et al., 2014). Learners have an active role in role playing method; this method can create hypothetical situations tailored to class circumstances. The role playing method facilitates the combination of theory and practice (Suen et al., 2011). Participants in this training method use all their senses (Chan, 2012). This method is suitable for changing the attitudes and individual's governing values and is a technique for discovering patients' information in the treatment process and training environment (Cushing & Jones, 1995). To date, many studies have been done regarding the effectiveness of different training methods in Iran and other countries. Among learning methods, role playing has been recognized as a superior method in enhancing learners' learning (Moradi & Didehban, 2017; Redden, 2015; Sato, Okamoto, Kayaba, Nobuhara, & Soeda, 2017). role playing has been designed as a training method of student‐centred to gain knowledge, attitude and skills in a wide spectrum of fields and learners in a safe and supportive environment (Acharya et al., 2014; Colletti, Gruppen, Barclay, & Stern, 2001).

Given the vital role of nurses in crisis situations and also the priority of the problems in pre‐hospitals cares, the paucity of information on triage or information multiplicity about standards and training needs of students and nurses in this field, this study aimed to compare the training effects on pre‐hospital triage using role playing and lecture methods in addition to examining the effectiveness of these two methods on knowledge, attitude and performance of nursing students in the context of pre‐hospital triage.

## 3 METHODS

### 3.1 Design

This pre‐test–posttest quasi‐experimental study examines and compares the effect of role playing and lecture on pre‐hospital triage in nursing students.

### 3.2 Sample and setting

**Figure 1:**
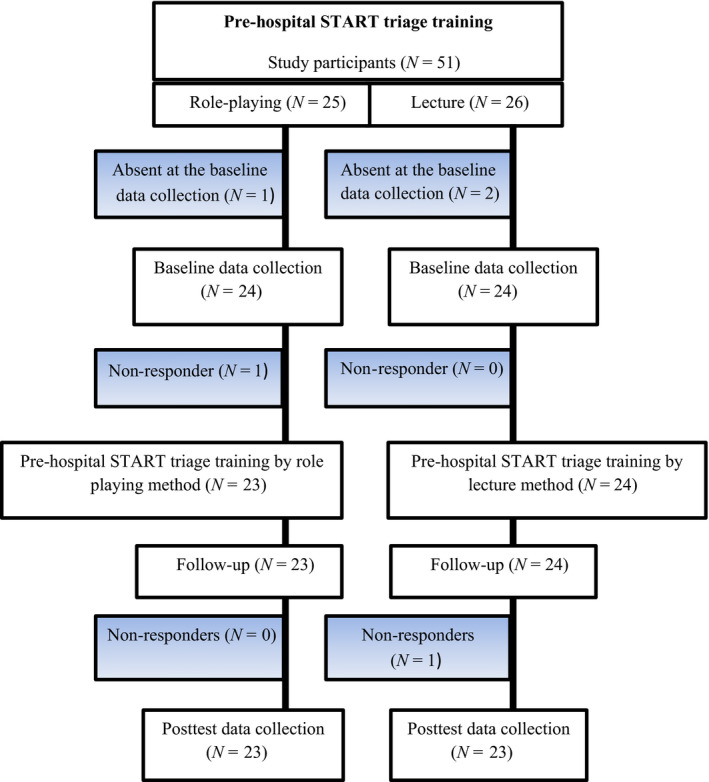
Flow chart of participants' enrolment for this study

The sample in this study included BSc nursing students of a university in the west of Iran who met the following criteria including (a) being 3rd year nursing students; (b) have not passed emergency and crisis course; (c) non‐attendance at workshops or educational classes of emergency and crisis; (d) having no work experience in emergency department or collaboration with organizations like Red Crescent, which provide emergency services; and (e) willing to participate in the study. With a 95% of confidence interval, power of 80% and *α* = .05, a sample size of 42 was determined for the present study. Considering sample attrition, all 3rd year BSc nursing students in semester 6 (*N* = 51) were selected (Figure 1). Exclusion criteria were as follows: failure to complete the questionnaire and non‐attendance in educational sessions in each stage of the study.

### 3.3 Intervention

After declaring the study objective, all students who met the including criteria invited to take part in the study voluntarily and signed an informed consent. Next, the pre‐test was done using study instruments. Before the random allocation, a 2‐hr session was held and START pre‐hospital triage concepts were taught to all students. Next, the students were matched based on GPA and then randomly assigned into two groups, the control group (lecture) and intervention group (role playing). In the lecture group, training process according to the content of START pre‐hospital triage was provided in three sessions, each session within a week, with 90 min dedication for each session, which was given in a PowerPoint format using lecture method. Intervention in the experimental group was carried out using the role playing method in three sessions according to the content of START pre‐hospital triage. Each session was held within a week, with 90 min dedication for each session. By the researcher, students were given designed scenarios of the possible role of rescuer nurse. Rescuer students by having triage cards and marker and other essentials materials in START triage system triaged presumptive injured people using scenarios where the situation of injured people was ascertained in a simulated environment. It is worth noting that the previously taught topics were reviewed and students' questions were answered at the beginning of each session. Immediately before the beginning of the intervention and 4 weeks after the last training session, the knowledge and attitude questionnaires were completed by students and samples' performance was assessed by a researcher‐made performance checklist.

### 3.4 Instruments

The questionnaire comprised of two sections. The first section was related to socio‐demographic characteristics and included age, gender, the GPA of six previous semesters and written GPA of diploma. In the second section of the questionnaire, the knowledge and attitude of participants were assessed. Knowledge and attitude were assessed using a questionnaire developed and validated by Haghdoust, Safavi, and Yahyavi (2009). Knowledge questionnaire consisted of 37 four‐choice questions that the score of 1 and 0 was given to right and wrong answer, respectively. So, the minimum score was 0, and the maximum score was 37. A score of <12.3 was considered as poor knowledge, score between 12.4–24.6 was considered as moderate, and score above 24.7 was considered as good knowledge. Attitude questionnaire comprised of 9 items, and each item scored on a visual scale 0–100. Therefore, the minimum score was 0, and the maximum score was 900. A score of <300 was considered a poor, score between 301–600 was considered as a moderate, and score above 601 was considered as a good attitude. The performance was examined using a 10 items checklist developed by research team based on START protocol content (Benson, Koenig, & Schultz, 1996; Gulli, Ciatolla, Barnes, & American Academy of Orthopaedic Surgeons, 2011). Each of checklist items was a scenario that adapted from START pre‐hospital triage. In each scenario, the participants performance was examined based on five main component of START pre‐hospital triage practices included priority, breathing positioning, capillary refill time and consciousness level. In each of five main components of START, the students' performance was assessed by two modes: true, 1; false or failure to do, 0.

Since the checklist consisted of 10 items and five components was measured in each item, the minimum and maximum scores were 0–50, respectively. A score of <16.6 was considered as poor, score between 16.7–33.3 was considered as moderate, and score above 33.4 was considered as a good performance.

The validity of the questionnaires was determined using the content validity method. In this regard, the questionnaires and checklist were given to 12 experts including three emergency medicine faculty member, 6 nursing faculty member and three emergency medical staff to evaluate the content of questionnaire. After receiving their comments, the necessary changes were made to the questionnaire. The reliability of questionnaires and checklist was assessed after a pilot study on 20 nursing students using Cronbach's alpha. The reliability was calculated to .91 and .86 for the knowledge and attitude scales, respectively. The inter‐rater reliability of performance checklist was assessed using inter‐rater reliability. There was no significant difference between the scores of two rater (*p* > .05), and the Cronbach's alpha of the scale for the score given by both observer was above .8.

### 3.5 Ethical consideration

All ethical considerations including obtaining permission from the Ilam University of Medical Sciences Ethics Committee (IR.MEDILAM.REC.1398.207), signed informed consent, protecting recorded information and observing the trusteeship about the use of resources were carefully considered in this study.

### 3.6 Statistical analysis

The data were analysed using SPSS version 22 (SPSS Inc.). Descriptive statistics such as per cent, mean and standard deviation were used summarize demographic and clinical characteristics of study participants. Independent and paired *t* tests were used to examine experiment and control group differences (knowledge, attitude and performance) from baseline to posttests. Chi‐squared test was used to examine demographic variables such as age and marital status between experiment and control groups. Alpha was set at .05.

## 4 RESULTS

A total of 51 participants were studied, of whom two and three participants from the role playing and lecture groups were excluded due to absenteeism in the data collection or pre‐hospital START triage training sessions, respectively. Finally, the data of 46 participants consisting of 23 nursing students in each of groups were analysed. The socio‐demographic characteristics of participants are shown in Table 1. As shown in Table 1, the mean (*SD*) of age of participants in the intervention group and the control group was 21.86 (*SD* 0.81) and 22.00 (*SD* 1.34), respectively. There was no significant difference between individual and social socio‐demographic characteristics of participants in the two groups (*p* > .05).

**Table 1:** Characteristics of participants in lecture and role playing groups

Variables	Lecture (*N* = 23)		role playing (*N* = 23)		*p*‐Value
	Mean (*SD*)	*N* (%)	Mean (*SD*)	*N* (%)	
Age	22.00 ± 1.34		21.86 ± 0.81		.79
Gender					
Male		14 (60.9)		11 (47.8)	.55
Female		9 (39.1)		12 (52.2)	
Marital status					
Married		2 (8.7)		3 (13.0)	1.00
Single		21 (91.3)		20 (87.0)	
GPA^a^ of 5 previous semesters	16.10 ± 0.96		16.52 ± 1.07		.16
GPA of high school	17.40 ± 1.08		17.83 ± 0.83		.13

a The GPA Score range in Iran is 0–20.

The mean of student knowledge and attitude for both groups before and after the START pre‐hospital triage training is shown in Table 2. As shown in Table 2, there was no significant difference between the mean (*SD*) difference of knowledge and attitude scores in the experimental group and the control group before and after the intervention (*p* > .05). Besides, according to the paired *t* test there was a significant difference in the knowledge and attitude of experimental group before and after the intervention (*p* < .001). There was also a significant difference in the knowledge and attitude of control group before and after the intervention (*p* < .05).

**Table 2:** Comparing the student knowledge and attitude in the intervention and the control groups before and after the START pre‐hospital triage training

Dependent variables	Lecture (*N* = 23)		role playing (*N* = 23)		*p*‐Value
	Baseline Mean (*SD*)	4 weeks Mean (*SD*)	Baseline Mean (*SD*)	4 weeks Mean (*SD*)	
Knowledge	15.30 ± 2.20	25.30 ± 3.37**	15.65 ± 2.53	26.52 ± 3.07**	
Change from baseline to follow‐up	10.00 ± 3.91		10.86 ± 4.56		.49
Attitude	685.43 ± 103.84	756.34 ± 58.22*	697.17 ± 89.40	808.60 ± 64.37**	
Change from baseline to follow‐up	70.91 ± 132.23		107.95 ± 101.76		.293

* *p* < .05 compared with baseline within the group.

** *p* < .001 compared with baseline within the group.

The mean of participants performance score for both groups before and after the START pre‐hospital triage training is shown in Table 3. As shown in Table 3, The mean (*SD*) difference of total performance score from baseline to follow‐up in the experimental group and the control group was 23.91 (13.83) and 7.00 (13.20), respectively, which indicates the total performance score in the experimental group improved significantly compared with the control group (*p* < .001). In three components of START pre‐hospital triage including priority, positioning and consciousness level, significant difference was observed between the mean difference of the experimental group and the intervention group before and after the intervention (*p* < .05). Besides, there was a significant difference in all components of START pre‐hospital triage in the experimental group before and after the intervention (*p* < .05). There was also a significant difference in priority, positioning and total score components of START pre‐hospital triage of control group before and after the intervention (*p* < .05).

**Table 3:** Comparing the student performance in the intervention and the control groups before and after the START pre‐hospital triage training

Components of START pre‐hospital triage	Control (*N* = 23)		Experiment (*N* = 23)		*p*‐Value
	Baseline Mean (*SD*)	12 weeks Mean (*SD*)	Baseline Mean (*SD*)	12 weeks Mean (*SD*)	
Priority	2.43 ± 1.44	4.34 ± 2.85*	2.17 ± 1.64	8.65 ± 2.53**	
Change from baseline to follow‐up	1.91 ± 3.28		6.47 ± 3.24		<.001
Breathing	3.52 ± 4.60	5.73 ± 2.87	3.21 ± 3.38	8.39 ± 2.70**	
Change from baseline to follow‐up	2.21 ± 6.01		5.11 ± 4.85		.073
Positioning	1.43 ± 1.87	3.82 ± 4.44*	1.56 ± 1.16	8.08 ± 3.50**	
Change from baseline to follow‐up	2.39 ± 5.16		6.52 ± 3.97		.004
Capillary refill time	4.37 ± 2.01	5.30 ± 3.09	4.56 ± 2.23	7.43 ± 3.11*	
Change from baseline to follow‐up	0.95 ± 3.52		2.86 ± 4.52		.117
Consciousness level	5.04 ± 3.44	4.56 ± 2.72	3.65 ± 2.47	6.52 ± 4.03*	
Change from baseline to follow‐up	−0.47 ± 3.81		2.86 ± 5.13		.016
Total performance score	16.78 ± 9.29	23.78 ± 8.08*	15.17 ± 8.51	39.08 ± 8.12**	
Change from baseline to follow‐up	7.00 ± 13.20		23.91 ± 13.83		<.001

* *p* < .05 compared with baseline within the group.

** *p* < .001 compared with baseline within the group.

## 5 DISCUSSION

This study aimed to determine and compare the effectiveness of two methods of role playing and lecture on nursing students' knowledge, attitude and performance in pre‐hospital triage. The results showed that the scores of knowledge, attitude and performance of nursing students increased in both groups. The scores of performance improved significantly in the role playing group than the lecture group before and after the intervention. To contrast, there was no significant difference in the score of knowledge and attitude in both groups despite a substantial increase before and after the intervention. No studies have been found concerning the comparison of the effectiveness of two methods of role playing and lecture in pre‐hospital triage on nursing students, according to the existing articles in databases such as Pub Med, Cochrane library, Scopus, CINAHL and Google Scholar. Delnavaz et al. (2018) found that both methods of lecture and role playing lead to an increase in knowledge and practice of nursing students in emergency severity index (ESI) triage. They also found that role playing method was more effective for triage education compared with lecture method (Delnavaz et al., 2018), which is in line with our results. In a study by Haghdoost et al., knowledge, attitude and practice of nursing students in pre‐hospital triage increased using the lecture method (Haghdoust et al., 2009).

Besides, a study which was conducted by Faraji, Khankeh, Hosseini, Abdi, and Rezasoltani (2013) is compatible with the results of this study. They showed that nurses' preparedness in the scenario group increased significantly compared with those of the lecture group in addition to increased preparedness in both groups after the intervention. The study by Hutchinson et al. (2011) showed the effectiveness of preparedness of role playing method in START pre‐hospital triage in unpredictable events among nursing students. Kim (2018) in a study examined the effect of simulation method on nursing students' self‐efficacy and critical thinking skills in emergency cardiac arrest and showed that students in the role playing method have a deeper understanding about the clinical setting and critical thinking skills in realistic situations in comparison with the lecture method.

Our study showed no significant difference between the groups of role playing and lecture despite the considerable increase in knowledge and attitude's score of nursing students after the intervention. Delnavaz et al. (2018) showed that the extent of knowledge is the same somewhat in role playing and lecture groups, but this was not in line with the study of Vizeshfar, Zare, and Keshtkaran (2019) which was conducted with the aim of comparing the two methods of role playing and lecture method in promotion of breastfeeding, this is possibly due to the nature of the content provided in different learning levels in two studies. The content of START pre‐hospital triage is a specific clinical protocol with easy understanding and slight volume, so there is no need for modern teaching method for the learning in the cognitive field of START pre‐hospital triage and only the lecture method is enough in this field.

Furthermore, given the different learning levels as well as various training methods used, it can be said that lecture method is a suitable method for learning in cognitive level and for learning in levels higher than cognitive domain including attitude and performance the modern training methods such as role playing should be used. Another possible reason for no significant difference in knowledge variable in the two training methods was that since lecture method was unable to enhance students' performance in pre‐hospital triage compared with role playing, the students' mind concentrated more on the cognitive domain during the three lecture sessions and students had a more opportunity for increasing learning in the cognitive domain through a workshop. In doing so, Cushing and Jones (1995) showed that role playing method is more suitable for change in attitudes and individual's governing values. Further, given that the role playing creates a safe and no stress environment to gain experience in a clinical setting, this is a suitable method for improving performance (Liebrecht & Montenery, 2016). Hence, it might be concluded that role playing method is an appropriate method for the learning in both attitude and practice domains. Also, considering that students have not been the experience use of modern teaching methods so far, the researcher observed students' interaction, motivation and satisfaction in the role playing group, which this might be attributed to the students' unwillingness to traditional methods repetition.

### 5.1 Limitations

This study conducted on junior‐level nursing students, so generalizing the results must be done with precaution for students of other levels and nurses employed. Besides, the limitation of the study duration due to executive limitations was not allowed the students' reminding to be assessed for long‐term learning retention. Hence, a longitudinal study is suggested for measuring learning retention.

## 6 CONCLUSION

The results showed that both of training methods lead to the promotion of students' learning in pre‐hospital triage. However, the role playing method is an attractive and suitable method for learning in the levels of knowledge, attitude and practice. The role playing method causes students' motivation and satisfaction of the learning experience. Furthermore, the role playing method is implementable with minimum facilities and costs in comparison with other simulation methods. Nursing educators can put in place the lecture method alongside other training methods as a complementary method for initial learning (cognitive) levels for nursing students.

## CONFLICT OF INTERESTS

We have no conflict of interest to declare.

## AUTHOR CONTRIBUTION

AA, ZH and HH involved in conception and design, drafting of the manuscript, and final approval of the manuscript HH and ZH involved in acquisition of data. AA and HH involved in analysis and interpretation of data.

## References

[nop2464-bib-0001] Acharya, S. , Shukla, S. , Acharya, N. , Vagha, J. , & Vagha, J. (2014). Role play‐an effective tool to teach clinical medicine. Journal of Contemporary Medical Education, 2(2), 91–96.

[nop2464-bib-0002] Aghababaeian, H. , Araghi Ahvazi, L. , Moosavi, A. , Ahmadi Mazhin, S. , Tahery, N. , Nouri, M. , … Kalani, L. (2019). Triage live lecture versus triage video podcast in pre‐hospital students' education. African Journal of Emergency Medicine, 9(2), 81–86. 10.1016/j.afjem.2018.12.001 31193815 PMC6543081

[nop2464-bib-0003] Atashzadeh shorideh, F. , Nikravan Moofrad, M. & Zohri Anbouhi, S. (2007). Triage, first aid and transporting of visitim (1st ed., pp. 10–45). Tehran, Iran: Noordanesh Publishers.

[nop2464-bib-0004] Bahrami, M. , Aliakbari, F. , & Aein, F. (2014). Investigation of competencies of nurses in disaster response by utilizing objective structured clinical examination. Iranian Journal of Nursing and Midwifery Research, 19(7 Suppl 1), S1–S6. 25949243 PMC4402988

[nop2464-bib-0005] Benson, M. , Koenig, K. L. , & Schultz, C. H. (1996). Disaster triage: START, then SAVE–a new method of dynamic triage for victims of a catastrophic earthquake. Prehospital and Disaster Medicine, 11(2), 117–124. 10.1017/S1049023X0004276X 10159733

[nop2464-bib-0006] Bux, A. (2009). Nurses' perceptions of the usefulness of high fidelity simulation technology in a clinical education program. Phoenix, AZ: University of Phoenix.

[nop2464-bib-0007] Chan, Z. C. (2012). role playing in the problem‐based learning class. Nurse Education in Practice, 12(1), 21–27. 10.1016/j.nepr.2011.04.008 21601528

[nop2464-bib-0008] Colletti, L. , Gruppen, L. , Barclay, M. , & Stern, D. (2001). Teaching students to break bad news. American Journal of Surgery, 182(1), 20–23. 10.1016/S0002-9610(01)00651-1 11532409

[nop2464-bib-0009] Cushing, A. , & Jones, A. (1995). Evaluation of a breaking bad news course for medical students. Medical Education, 29(6), 430–435. 10.1111/j.1365-2923.1995.tb02867.x 8594407

[nop2464-bib-0010] Delnavaz, S. , Hassankhani, H. , Roshangar, F. , Dadashzadeh, A. , Sarbakhsh, P. , Ghafourifard, M. , & Fathiazar, E. (2018). Comparison of scenario based triage education by lecture and role playing on knowledge and practice of nursing students. Nurse Education Today, 70, 54–59. 10.1016/j.nedt.2018.08.006 30145535

[nop2464-bib-0011] Drobatz, K. J. , Hopper, K. , Rozanski, E. A. , & Silverstein, D. C. (2018). Textbook of small animal emergency medicine (pp. 4–6). Hoboken, NJ: Wiley‐Blackwell.

[nop2464-bib-0012] Ebrahimi, M. , Mirhaghi, A. , Mazlom, R. , Heydari, A. , Nassehi, A. , & Jafari, M. (2016). The role descriptions of triage Nurse in Emergency department: A Delphi Study. Scientifica (Cairo), 2016, , 1–6. 10.1155/2016/5269815 PMC4921622 27382500

[nop2464-bib-0013] Faraji, A. , Khankeh, H. , Hosseini, M. , Abdi, K. , & Rezasoltani, P. (2013). Effect of simulated training course on preparedness of nurses to do pre‐hospital triage. Journal of Health Promotion Management, 2(4), 24–29.

[nop2464-bib-0014] Frenk, J. , Chen, L. , Bhutta, Z. A. , Cohen, J. , Crisp, N. , Evans, T. , … Zurayk, H. (2010). Health professionals for a new century: Transforming education to strengthen health systems in an interdependent world. Lancet, 376(9756), 1923–1958. 10.1016/S0140-6736(10)61854-5 21112623

[nop2464-bib-0015] Gulli, B. , Ciatolla, J. A. , Barnes, L. , & American Academy of Orthopaedic Surgeons (2011). Emergency care and transportation of the sick and injured. Burlington, MA: Jones and Bartlett.

[nop2464-bib-0016] Haghdoust, Z. , Safavi, M. , & Yahyavi, H. (2009). Effect of triage education on knowledge, attitude and practice of nurses in Poursina Educational and Therapeutic Emergency Center in Rasht. Guilan Journal of Nursing and Midwifery, 20(64), 14–21. In Persian.

[nop2464-bib-0017] Heidarzadeh, H. , Hassankhani, H. , Dadashzadeh, A. , Fathi‐Azar, E. , Moghadasian, S. , & Haririan, H. (2017). Pre‐hospital Triage: Knowledge, readiness and performance of nursing students in dealing with unexpected accidents. Iranian Journal of Emergency Care, 1(2), 46–55.

[nop2464-bib-0018] Hutchinson, S. W. , Haynes, S. , Parker, P. , Dennis, B. , McLIN, C. , & Welldaregay, W. (2011). Implementing a multidisciplinary disaster simulation for undergraduate nursing students. Nursing Education Perspectives, 32(4), 240–243. 10.5480/1536-5026-32.4.240 21923004

[nop2464-bib-0019] Khajeahmadi, M. , & Jahanpour, F. (2017). Investigating the privacy practices of patients among trainees and interns of the Faculty of Nursing and Midwifery of Bushehr University of Medical Sciences in 1395. Journal of Medical Ethics and History of Medicine, 10(1), 141–154.

[nop2464-bib-0020] Kim, E. (2018). Effect of simulation‐based emergency cardiac arrest education on nursing students' self‐efficacy and critical thinking skills: Roleplay versus lecture. Nurse Education Today, 61, 258–263. 10.1016/j.nedt.2017.12.003 29274573

[nop2464-bib-0021] Liebrecht, C. , & Montenery, S. (2016). Use of simulated psychosocial role playing to enhance nursing students' development of soft skills. Creative Nursing, 22(3), 171–175. 10.1891/1078-4535.22.3.171 29195526

[nop2464-bib-0022] Lyles, D. C. (2009). Use of human patient simulators and perceived self‐efficacy of nursing skills in associate degree nursing students. [Ph.D.]. Ann Arbor, MI: Capella University.

[nop2464-bib-0023] Maddry, J. K. , Varney, S. M. , Sessions, D. , Heard, K. , Thaxton, R. E. , Ganem, V. J. , … Bebarta, V. S. (2014). A comparison of simulation‐based education versus lecture‐based instruction for toxicology training in emergency medicine residents. Journal of Medical Toxicology, 10(4), 364–368. 10.1007/s13181-014-0401-8 24844460 PMC4252281

[nop2464-bib-0024] Mahmoodian, H. , Eghtesadi, R. , Ghareghani, A. , & Nabeiei, P. (2016). Knowledge of triage in the senior medical students in Shiraz University of Medical Sciences. Journal of Advances in Medical Education and Professionalism, 4(3), 141–144. 27382582 PMC4927257

[nop2464-bib-0025] Moradi, E. , & Didehban, H. (2017). Requirements for the proper use of role playing methods at medical universities. Teb Va Tazkiye, 25(3), 147–156.

[nop2464-bib-0026] Naderifar, M. , Ghaljaei, F. , Jalalodini, A. , Rezaie, N. , & Salar, A. (2016). Challenges of e‐learning in medical sciences: A review article. Journal of Medical Education Development, 9(23), 102–111.

[nop2464-bib-0027] Redden, S. L. (2015). The effectiveness of combining simulation and role playing in nursing education [Ed.D.]. Ann Arbor, MI: Walden University.

[nop2464-bib-0028] Sabounchi, S. S. , Sabounchi, S. S. , & Safari, M. (2019). Knowledge and attitude of midwifery students on oral health care. Dentistry Journal, 7(3), 83 10.3390/dj7030083 PMC6784477 31374979

[nop2464-bib-0029] Sato, Y. , Okamoto, S. , Kayaba, K. , Nobuhara, H. , & Soeda, K. (2017). Effectiveness of role‐play in hazard prediction training for nursing students: A randomized controlled trial. Journal of Nursing Education and Practice, 8, 1–7. 10.5430/jnep.v8n2p1

[nop2464-bib-0030] Sedaghat, S. , Aghababaeian, H. , Taheri, N. , Sadeghi, M. A. , Maniey, M. , & Araghi, A. L. (2012). Study on the level of knowledge and performance of North Khuzestan medical emergency 115 personnel on pre‐hospital triage. Iranian Journal of Critical Care Nursing, 5(2), 103–108.

[nop2464-bib-0031] Suen, W. , Hughes, J. , Russell, M. , Lee, H. , Carr, A. , Parker, V. , & Rao, S. (2011). From role play to real play: teaching effective role playing facilitation skills. MedEdPORTAL Publications, 7, 8603 10.15766/mep_2374-8265.8603

[nop2464-bib-0032] Tuyisenge, L. , Kyamanya, P. , Van Steirteghem, S. , Becker, M. , English, M. , & Lissauer, T. (2014). Knowledge and skills retention following Emergency Triage, Assessment and Treatment plus Admission course for final year medical students in Rwanda: A longitudinal cohort study. Archives of Disease in Childhood, 99(11), 993–997. 10.1136/archdischild-2014-306078 24925893 PMC4198299

[nop2464-bib-0033] Vizeshfar, F. , Zare, M. , & Keshtkaran, Z. (2019). Role‐play versus lecture methods in community health volunteers. Nurse Education Today, 79, 175–179. 10.1016/j.nedt.2019.05.028 31136868

